# Lin28a forms an RNA‐binding complex with Igf2bp3 to regulate m^6^A‐modified stress response genes in stress granules of muscle stem cells

**DOI:** 10.1111/cpr.13707

**Published:** 2024-07-17

**Authors:** Dan Song, Yu Chen, Peng Wang, Yeqian Cheng, Ng Shyh‐Chang

**Affiliations:** ^1^ Key Laboratory of Organ Regeneration and Reconstruction, State Key Laboratory of Stem Cell and Reproductive Biology Institute of Zoology, Chinese Academy of Sciences Beijing China; ^2^ Institute for Stem Cell and Regeneration Chinese Academy of Sciences Beijing China; ^3^ University of Chinese Academy of Sciences Beijing China; ^4^ Beijing Institute for Stem Cell and Regenerative Medicine Beijing China

## Abstract

In the early embryonic stages, Lin‐28 homologue A (Lin28a) is highly expressed and declines as the embryo matures. As an RNA‐binding protein, Lin28a maintains some adult muscle stem cells (MuSCs) in an embryonic‐like state, but its RNA metabolism regulation mechanism remains unclear. BioGPS analysis revealed that Lin28a expression is significantly higher in muscle tissues than in other tissues. Lin28a‐positive muscle stem cells (Lin28a+ MuSCs) were sorted from *Lin28a‐CreERT2*; *LSL‐tdTomato* mouse skeletal muscle tissue, which exhibited a higher proliferation rate than the control group. Lin28a‐bound transcripts are enriched in various biological processes such as DNA repair, cell cycle, mitochondrial tricarboxylic acid cycle and oxidative stress response. The expression of insulin‐like growth factor 2 mRNA‐binding protein 3 (Igf2bp3) was markedly elevated in the presence of Lin28a. Co‐immunoprecipitation analysis further demonstrated that Lin28a associates with Igf2bp3. Immunofluorescence analyses confirmed that Lin28a, Igf2bp3 and G3bp1 colocalize to form stress granules (SG), and N6‐methyladenosine (m^6^A) modification promotes the formation of Lin28a‐SG. Sequencing of the transcriptome and RNAs immunoprecipitated by Lin28a, Igf2bp3 and m^6^A antibodies in Lin28a+ MuSCs further revealed that Lin28a and Igf2bp3 collaboratively regulate the expression of DNA repair‐related genes, including *Fancm* and *Usp1*. Lin28a stabilises *Igf2bp3*, *Usp1,* and *Fancm* mRNAs, enhancing DNA repair against oxidative or proteotoxic stress, thus promoting MuSCs self‐renewal. Understanding the intricate mechanisms through which Lin28a and Igf2bp3 regulate MuSCs provides a deeper understanding of stem cell self‐renewal, with potential implications for regenerative medicine.

## Graphical abstract

Lin‐28 homologue A (Lin28a) controls the expression of genes involved in DNA damage repair via insulin‐like growth factor 2 mRNA‐binding protein 3 (Igf2bp3) and enhances the DNA stress response of muscle stem cells (MuSCs) by regulating N6‐methyladenosine‐modified mRNAs within stress granules. This positive regulation contributes to the proliferation and self‐renewal of MuSCs.

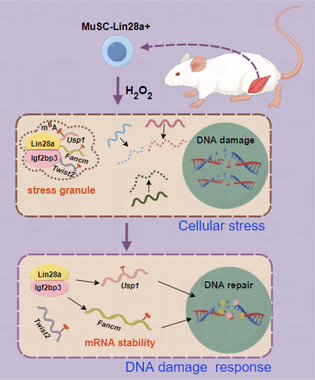

## 1 INTRODUCTION

Skeletal muscle is composed of aligned bundles of myofibers, complex vasculature and lymphatic vessels, which are regulated by a network of nerves and the extracellular matrix (ECM).^1^ Muscle stem cells (MuSCs) are a class of somatic stem cells, also known as satellite cells, that normally remain in a quiescent state in skeletal muscle. MuSCs play an important role in muscle repair, proliferation, differentiation and eventually the formation of new muscle tissue after muscle injury.^2^, ^3^ However, the accumulation of injuries can also lead to a decrease in the number and diminished function of MuSCs, eventually producing diseases such as muscle loss or muscle insufficiency.^4^, ^5^ For example, MuSCs may encounter stress stimuli under various conditions, including muscle injury or trauma, inflammation and oxidative stress.^6^, ^7^

Oxidative stress is a common physiological occurrence that results from an imbalance in cellular redox states, leading to the generation of reactive oxygen species. In conditions such as inflammation or chronic diseases, oxidative stress can escalate, causing cell cycle arrest, DNA damage and apoptosis.^8^ Thus, a comprehensive understanding of the oxidative stress mechanism affecting MuSCs is crucial for preventing muscle ageing and treating various diseases of muscle dysfunction.

Lin‐28 homologue A (Lin28a), as an RNA‐binding protein, is shown to bind precursor forms of *let‐7* miRNAs, inhibiting pri‐let‐7 processing by Drosha.^9^ Therefore, Lin28a regulates the expression of multiple target genes by inhibiting *let‐7* maturation.^10^, ^11^, ^12^ Lin28a also has been shown to promote the pluripotent state of embryonic stem cells (ES) and induced pluripotent stem cells (iPS).^13^ In addition, Lin28a has been implicated in the regulation of glucose metabolism and tissue regeneration.^14^, ^15^ Insulin‐like growth factor 2 mRNA‐binding protein 3 (Igf2bp3), also an RNA‐binding protein, recognises N6‐methyladenosine (m^6^A) modifications and affects mRNA stability and subcellular localization, which plays a critical role in the post‐transcriptional regulation of gene expression.^16^ Additionally, Igf2bp3 facilitates the transport of target mRNAs to specific subcellular locations, such as stress granules (SG) and P‐bodies, where they can be stored or undergo translational repression.^17^, ^18^

RNA metabolism involves processes such as transcription, splicing, RNA modification and RNA degradation. It is tightly regulated to ensure proper gene expression and cellular function.^19^, ^20^ Stress granules are dynamic cytoplasmic structures that form in response to various stresses, including heat shock, oxidative stress and viral infections.^21^ They contain RNA‐binding proteins (RBPs), translation initiation factors and other regulators of RNA metabolism. Stress granules can sequester mRNAs, halt their translation and modulate RNA stability and turnover, to prepare the cell to mount a series of cellular stress responses.^22^, ^23^, ^24^ However, it has remained unclear how SG regulates the expression of DNA repair genes, if any.

As previously reported in our research, Lin28a maintains a subset of adult MuSCs in an embryonic‐like state.^25^ How does Lin28a maintain cell rejuvenation through the regulation of RNAs? Here, we analysed the expression of Lin28a in different developmental stages using the public database BioGPS and found that its expression is low in most adult differentiated tissues, but highest in muscle tissues. Through bioinformatics analysis and experimental validation, we found that the Lin28a‐Igf2bp3 complex regulates a large number of m^6^A‐modified RNAs, particularly mRNAs related to mitosis, cellular metabolism and DNA damage repair. When MuSCs encounter stress stimuli, the m^6^A‐modified RNAs regulated by the Lin28a‐Igf2bp3 complex are preferentially stored in SG to ensure higher mRNA stability and are routed for protein translation. This preferential regulation serves to maintain the DNA stress response and genomic stability of MuSCs, thereby ensuring their capacity for self‐renewal and proliferation.

## 2 MATERIALS AND METHODS

### 2.1 Animals

All animal procedures were approved by the Institute of Zoology and the Institute of Stem Cell and Regenerative Medicine, Chinese Academy of Sciences (IOZ‐IACUC‐2022‐170). The mice were provided with regular chow and housed in a controlled environment with a 12:12‐h light–dark cycle at 22°C.

### 2.2 Cell lines and culture conditions

MuSCs were cultured as previously described.^25^ The culture plates were first coated with 1% Matrigel (Corning, 354230).

### 2.3 siRNAs, plasmids and antibodies

To silence target genes, cells were transfected with small interfering RNAs (siRNAs, 10 nM), including siIgf2bp1: 5′‐CGACCAAGUCAUUGUUAAGdTdT‐3′/5′‐GAAACACCUGACUCCAAAGdTdT‐3′, siIgf2bp2:5′‐CCGUUGUCAACGUCACCUAdTdT‐3′/5′‐CUGUACCCUCAUCACCAUUdTdT‐3′, siIgf2bp3: 5′‐CGCGGAGAAGUCCAUUACUAUdTdT‐3′/5′‐CCUUAGACAAACUGAAUGGAUdTdT‐3′, siLin28a: 5′‐AUGUUCUUCCCUUUUGGCCdTdT‐3′/5′‐GGCCAAAAGGGAAGAACAUdTdT‐3′, siTut4: 5′‐GAGGAAAUGUCAAAGGUUAdTdT‐3′/5′‐GCAGCUAUUGAUCCUAGAGdTdT‐3′, siTut7: 5′‐GCGGCCUUCUUUGUAAAGUdTdT‐3′/5′‐AUGACAGGUGCUGCCGAAUdTdT‐3′.

To overexpress Lin28a, the cDNA sequence of Lin28a was inserted into the pCDH‐EF1‐EGFP‐T2A‐puro plasmid, and then packaged to produce lentivirus for infection of MuSCs. Antibodies, including Lin28a (8641s) and p‐H2AX (2577s), were purchased from Cell Signalling Technology. Igf2bp3 (A4444) and Usp1 (A6785) were purchased from Abconal. Gapdh (MA1‐16757) was purchased from Thermo Fisher Scientific. Twist2 (66544), Fancm (12954), G3bp1 (66486) and Tia1 (66907) were purchased from Proteintech. Igf2bp3 (sc‐390639) was purchased from Santa Cruz.

### 2.4 Immunofluorescence staining

MuSCs were plated on glass coverslips in a 12‐well plate to reach 80% confluence. The cells were treated with H_2_O_2_ (1 mM, 10 min) or NaAsO_2_ (200 μM, 2 h) to produce stress granule. Rinse briefly in phosphate‐buffered saline (PBS). The cells were fixed using 4% paraformaldehyde in PBS at pH 7.4 for 15 min at room temperature and then washed three times with ice‐cold PBS. Cells were treated with 0.25% Triton X‐100 in PBS for 10 min at room temperature. After incubation, the cells were incubated with 1% goat serum in PBS for 1 h at room temperature to block non‐specific binding sites. After blocking, cells were incubated with primary antibodies specific to the target cellular components overnight at 4°C. Then, the cells were incubated with fluorescently labelled secondary antibodies for 2 h at room temperature. Finally, cells were stained with 4′,6‐diamidino‐2‐phenylindole for nuclear visualisation, mounted on glass slides and observed and analysed using a laser‐scanning confocal microscope (Leica SP8).

### 2.5 Co‐immunoprecipitation and Western blot

MuSCs were lysed in 1× cell lysis buffer (CST, 9803) with the addition of protease inhibitors and RNase inhibitors, followed by centrifugation at 4°C and 12,000 rpm for 10 min. A 10% fraction of the supernatant was collected as the input sample, whilst the remaining supernatant was divided into two equal parts. One part was used as an IgG control, and the other part was subjected to protein complex pulldown using specific antibodies with protein A/G beads (Thermo, 78609). The validation of protein interactions was then performed through Western blot analysis. Proteins were separated by 12% sodium dodecyl sulfate‐polyacrylamide gel electrophoresis, and transferred from the gel onto a ployvinylidene fluoride membrane. The membrane was incubated with antibodies specific to the target protein. The results were analysed and quantified using imaging techniques (Thermo, FLI1000).

### 2.6 Cell cycle analysis by flow cytometry

MuSCs were harvested in 250 μL of PBS and added to 750 μL of anhydrous ethanol overnight at −20°C for fixation. Cells were collected by centrifugation and stained with a solution containing 50 μg/mL of propidium iodide, 50 μg/mL of RNase A and 3.8 mM of sodium citrate. The stained cells were passed through a flow cytometer (BD, FACSAria IIIu), and the fluorescence emitted by individual cells was measured. Finally, the number of cells distributed in the G1 (Gap 1), S (DNA synthesis), G2 (Gap 2) and M (mitosis) phases was counted and analysed.

### 2.7 RNA extraction and quantitative reverse transcription polymerase chain reaction (PCR)

MuSCs were collected in 1 mL of Trizol and left on ice for 5 min. Two‐hundred microliters of chloroform was added and mixed thoroughly. The cell samples were centrifuged at 12000 rpm for 10 min at 4°C. The supernatant was collected, and an equal volume of isopropanol was added, followed by incubation at −20°C for 2 h. Finally, the RNA was collected by centrifugation at 12,000 rpm for 10 min at 4°C, and the precipitate was washed with 80% ethanol, followed by dissolution in nuclease‐free water.

The extracted RNA was reverse transcribed into a cDNA library following the instructions provided with the reverse transcription kit (Takara, RR037A). The cDNA samples are mixed with SYBR Green master mix (YEASEN, 11201ES08), including specific primers targeting the gene of interest and reference genes for normalisation. In this study, the primers used for quantitative analysis of gene expression were listed in Table S1.

### 2.8 Methylated RNA immunoprecipitation

One‐hundred micrograms of total RNA was used to enrich mRNA using the Dynabeads™ mRNA Purification Kit (Thermo, 61006) according to the manufacturer's instructions. Ten percent of the enriched mRNA was used as input, and the remaining RNA was added to RNA Fragmentation Reagents (Invitrogen, AM8740). The mixture was incubated at 70°C in a PCR instrument for 10 min to fragment the RNA into approximately 100 nt fragments. The fragmented RNA was precipitated using an ethanol‐based method.

Washing protein A/G magnetic beads with immunoprecipitation (IP) buffer (150 mM NaCl, 10 mM Tris–HCl and pH 7.5), and then incubating them with 5 μg of m^6^A antibody (Millipore, ABE572) at 4°C for 2 h. Beads were washed twice with IP buffer and then resuspended in IP buffer. The fragmented RNA was added to the resuspended beads and the mixture was incubated at 4°C with gentle agitation for 4 h.

The beads with m^6^A complex were washed three times with IP buffer at 4°C, and treated with proteinase K. The remaining RNA was purified using a phenol:chloroform:isoamylalcohol (125:24:1) solution. Finally, the enriched RNA was precipitated and recovered using an ethanol‐based method. The enriched m^6^A‐modified RNA was used to construct a sequence library using an RNA‐seq Library Prep Kit (Vazyme, NR605), followed by sequencing analysis using Illumina HiSeq X10.

### 2.9 Bioinformatics analysis of high‐throughput sequencing

The raw data from RNA‐seq and RNA immunoprecipitation (RIP)‐seq were preprocessed by removing adapter sequences, filtering low‐quality reads and trimming low‐quality bases using FastQC. Subsequently, the data were aligned to the GRCh38 reference genome using Hisat2 (version 2.0.4). Differential gene expression analysis of the transcriptome was performed using DESeq2 (version 1.42.0) (*p* < 0.05). The differentially methylated m^6^A peaks were identified using MACS2 (version 1.4.3). Sequence motifs and annotations were analysed by Homer (version 4.11). The distribution of the m^6^A peaks was analysed using Guitar (version 2.18.0). Integrative Genomics Viewer (IGV, version 2.10.3) was used to visualise the peaks. ntrinsically disordered regions of Lin28a and Igf2bp3 were analysed using the online prediction tool IUPred2A by NovoPro. The expression analysis of Lin28a in various cells and tissues was obtained from BioGPS (dataset: GeneAtlas U133A, gcrma, expression data of human iPS, human ES and those differentiated cells [DC]).

### 2.10 RNA stability analysis

MuSCs‐control and MuSCs‐Lin28a+ were seeded in equal amounts in a 12‐well plate, with three replicates per group. After the cells adhered to the plate, they were treated with actinomycin D (10 μM), and cells were collected at 0, 1, 2, 3 and 4 h. RNA was extracted and reverse transcribed into cDNA, and the changes in mRNA levels of the target genes were detected by real‐time PCR. Linear regression was performed on the data of the target gene at each time point to determine the time required for each group to show mRNA decay to half of its initial level.

### 2.11 Live cell imaging analysis

MuSCs expressing GFP‐Lin28a were seeded into Cell Culture Imaging Dishes. After the cells completely adhered to the dish, 20 μM of the m^6^A inhibitor STM2457 (MedChemExpress, HY‐134836) was added and incubated for 24 h. Then, live‐cell imaging microscopy was used to record the process of stress granule formation from 0 to 15 min after adding 1 mM H_2_O_2_. The time and size of GFP‐Lin28a‐SG formation were measured under both STM2457 (METTL3 methyltransferase inhibitor) treated and untreated conditions.

### 2.12 Cell Counting Kit‐8 (CCK8) analysis

The expression levels of Lin28a and Igf2bp3 were knocked down in MuSCs using siRNAs for 24 h. The cells were plated in a 96‐well plate with 3000 cells per well and at least three replicates. The cells were treated with gradient concentrations of H_2_O_2_ or NaAsO_2_. After 24 h of treatment, replaced in the culture medium containing 10% CCK8 (APExBIO, K1018), then incubated at 37°C for 2 h. The absorbance of the cells was measured at 450 nm using a microplate reader.

### 2.13 Statistical analysis

Each experiment was performed with a minimum of three biological replicates to ensure statistical significance. Statistical analysis was performed using appropriate tests such as Student's *t*‐test or analysis of variance to determine the significance of differences between two groups. All statistical tests were two‐sided. *p*‐Value <0.05 was considered statistically significant. *p < 0.05, **p < 0.01, ***p < 0.001, ns, no significant.

## 3 RESULTS

### 3.1 Lin28a promotes MuSCs proliferation

**FIGURE 1:**
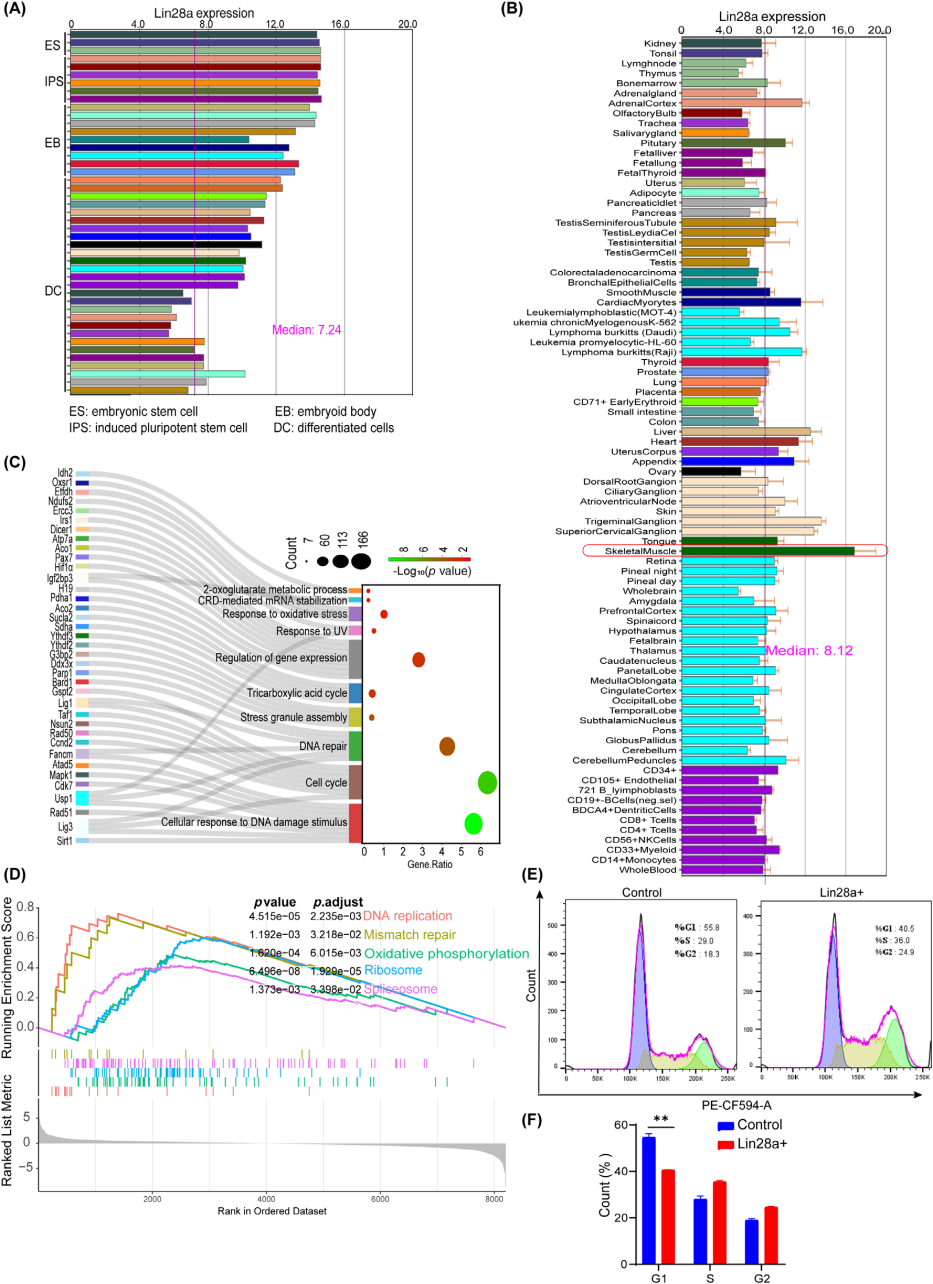
Lin‐28 homologue A (Lin28a) promotes muscle stem cells (MuSCs) proliferation by accelerating the G1 phase. (A) The mRNA expression levels of Lin28a were analysed in embryonic stem cells (ES), induced pluripotent stem cells (IPS), embryoid body (EB) and differentiated cells (DC) as the culture days increased, using the BioGPS database. (B) Expression pattern of Lin28a across various adult human tissues, with the highest expression level observed in skeletal muscle, according to the BioGPS database. (C) Gene ontology annotating Lin28a‐RIP data to analyse the biological processes involving Lin28a‐bound transcripts. (D) Performing Gene Set Enrichment Analysis to identify enriched signalling pathways in Lin28a+ versus control MuSCs. (E) Flow cytometric analysis was performed to examine the cell cycle of the Lin28a+ and control MuSCs. (F) Statistical analysis showed the proportions of cell cycle phases in each MuSCs population. Data are presented as means ± standard error of mean (SEM). Student's *t*‐test was performed for statistical analysis (**p < 0.01).

Lin28a, a crucial factor in maintaining cellular stemness, is important for embryonic stem cell maintenance, individual development and tissue differentiation. According to the analysis of the BioGPS public database,^26^ the highest expression of Lin28a was found in ES, followed by iPS and embryoid body cells, and the lowest level was found in DC (Figure 1A). However, we further analysed the microarray expression data of various tissues in the human body, and the results showed that Lin28a was much higher in muscle tissues than in other tissues (Figure 1B). Lin28a‐positive muscle stem cells (Lin28a+ MuSCs) were sorted from *Lin28a‐CreERT2*; *LSL‐tdTomato* mouse skeletal muscle tissue^25^ using flow cytometry. Lin28a‐RIP sequencing and transcriptome analysis were performed. Gene ontology (GO) annotation showed that transcripts bound by Lin28a are mainly enriched in biological processes such as DNA repair, cell cycle, mitochondrial tricarboxylic acid cycle (TCA cycle) and oxidative stress response (Figure 1C). Similarly, Gene Set Enrichment Analysis revealed significant differences in processes such as DNA repair, DNA replication and oxidative phosphorylation between the Lin28a+ and control MuSCs (Figure 1D). Furthermore, we analysed the m^6^A modification of MuSCs using methylated RNA immunoprecipitation (MeRIP) sequencing (Lin28+ vs. control). The results showed a positive correlation between m^6^A modification abundance and RNA expression level in MuSCs, with a Pearson's *R* value of 0.89 (Figure S1A). Heatmap analysis showed higher expression and m^6^A modification of transcripts involved in many biological processes such as DNA repair (Figure S1B), cell cycle (Figure S1C) and oxidative phosphorylation (Figure S1D), which indicates a more active metabolic state in Lin28a+ MuSCs. Flow cytometry‐based cell cycle analysis revealed that Lin28a+ MuSCs proliferated more rapidly by decreasing the duration of the G1 phase (Figure 1E,F). CCK8 analysis showed a higher proliferation rate for both Lin28a+ MuSCs and MuSCs overexpressing Lin28a (Figure S1E). When Lin28a expression was inhibited using siRNAs, the proliferation rate of MuSCs was reduced (Figure S1F). Overall, Lin28a promotes mitochondrial oxidative phosphorylation, the oxidative stress response, DNA damage repair and the proliferation of MuSCs.

### 3.2 Lin28a forms stress granules with Igf2bp3

**FIGURE 2:**
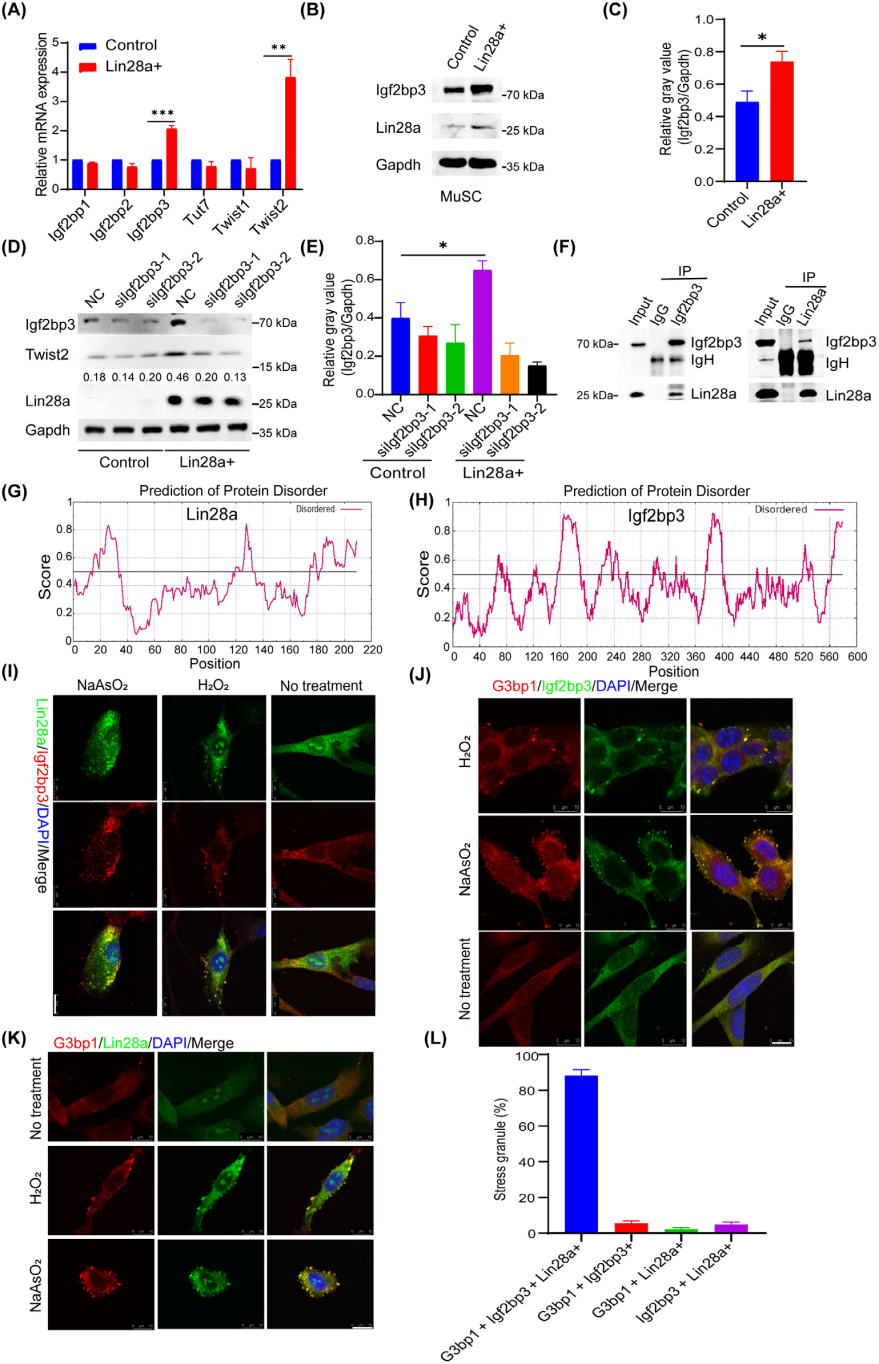
Lin‐28 homologue A (Lin28a) forms stress granules with insulin‐like growth factor 2 mRNA‐binding protein 3 (Igf2bp3). (A) RT‐qPCR analysis of the expression of Lin28a‐associated target genes between the Lin28a+ and control muscle stem cells (MuSCs) groups. (B) Western blot analysis revealed an increase in the protein level of Igf2bp3 when Lin28a was expressed in MuSCs. (C) Statistical analysis of the grey values of Igf2bp3/Gapdh. (D) Western blot analysis of Twist2 protein expression in Lin28a+ and control MuSCs groups after knockdown of Igf2bp3. (E) Statistical analysis of the grey values of Igf2bp3/Gapdh. (F) Co‐immunoprecipitation analysis was conducted in MuSC‐Lin28a+ cells to examine the interaction of Igf2bp3 and Lin28a. Intrinsically disordered regions of Lin28a (G) and Igf2bp3 (H) proteins were predicted using the IUPred2A. (I) Lin28a+ MuSCs were stimulated with oxidative stress using hydrogen peroxide (1 mM, 10 min), or oxidative and proteotoxic stress using sodium arsenite (200 μM, 2 h), and immunofluorescence analysis of the formation of stress granules by Igf2bp3 and Lin28a. Scale bar: 10 μm. (J) Immunofluorescence analysis of the formation of stress granules by Igf2bp3 and G3bp1. Scale bar: 10 μm. (K) Immunofluorescence analysis of the formation of stress granules by Lin28a and G3bp1. Scale bar: 10 μm. (L) Statistical analysis of the proportion of various types of stress granules. Data are presented as means ± SEM. Student's *t*‐test was performed for statistical analysis (*p < 0.05, **p < 0.01 and ***p < 0.001). DAPI, 4′,6‐diamidino‐2‐phenylindole; NC, negative control siRNA. RT‐qPCR, Quantitative reverse transcription polymerase chain reaction.

According to the expression analysis of Lin28a in BioGPS, we retrieved a list of its related genes with a correlation coefficient (*R*) greater than 0.5, as shown in Table S2. Through quantitative reverse transcription PCR (RT‐quantitative reverse transcription polymerase chain reaction (qPCR)) analysis in Lin28a+ MuSCs, we found that the expression levels of *Igf2bp3* and *Twist2* were significantly increased when Lin28a was highly expressed (Figure 2A). Western blot analysis also revealed higher levels of Igf2bp3 in Lin28a+ MuSCs (Figure 2B,C).

To investigate the functional relationships between Lin28a, Igf2bp3 and Twist2, we confirmed that overexpression of Lin28a in MuSCs increased the expression of Igf2bp3 and Twist2 through Western blot analysis. Additionally, when the expression of Igf2bp3 was knocked down using siRNAs, the expression of Twist2 decreased specifically in MuSCs that overexpressed Lin28a (Figure 2D,E), thus demonstrating the epistatic relationships in this regulatory pathway. Given that Twist2 has been previously shown to regulate stem cell proliferation and self‐renewal,^27^ we propose that Lin28a promotes MuSCs proliferation through a Lin28a‐Igf2bp3‐Twist2 pathway.

Given that both Lin28a and Igf2bp3 are RBPs, we asked if the two proteins also show direct protein–protein interactions. Reciprocal co‐immunoprecipitation analysis with either the Lin28a antibody or the Igf2bp3 antibody revealed that Lin28a protein does associate with Igf2bp3 protein in MuSCs as well (Figure 2F), and thus Lin28a promotes the formation of a Lin28a‐Igf2bp3 protein complex by autoregulating *Igf2bp3* expression (Figure 2B, C). Many RBPs that are known to interact with RNA, also form SG after exposure to stress‐inducing stimuli.^28^, ^29^ SG‐forming RBPs often contain intrinsically disordered regions (IDRs), which are regions of the protein that lack a well‐defined secondary or tertiary structure.^30^ IDRs are highly flexible and dynamic, allowing them to adopt multiple conformations and interact with a variety of binding partners. This flexibility is thought to be important for RBPs to interact with multiple RNA targets and to facilitate the assembly of SGs.^31^ For example, the IDR of the RBP TIA‐1 is required for its ability to phase separate and form SGs, whilst the IDR of G3BP1 is important for the recruitment of mRNAs to SGs.^32^, ^33^ Thus, we analysed the IDRs of Lin28a and Igf2bp3 using the online prediction tool IUPred2A (Figure 2G,H).^34^ Both Lin28a and Igf2bp3 exhibited significant numbers of IDRs, providing the basis for phase separation. In particular, Lin28a had one IDR at each terminus, flanking the cold shock domain (amino acids 39–112) and zinc finger domain (amino acids 137–176), respectively, suggesting that the RNA‐binding domains and their RNA targets would interact closely with the SG‐forming IDRs during a stress response. G3bp1 is a critical component in the assembly of SG, which are cellular structures formed in response to various stressors. These granules serve as sites for mRNA storage and regulation during stress conditions. G3bp1's interaction with other proteins, particularly RasGAP, facilitates stress granule formation and dynamics, influencing mRNA metabolism and translation regulation.^32^ We treated MuSCs with H_2_O_2_ (1 mM, 10 min) or NaAsO_2_ (200 μM, 2 h) to model cellular oxidative and proteotoxic stress stimuli. Immunofluorescence analyses confirmed that Lin28a, Igf2bp3 and G3bp1 colocalized to form SGs (Figure 2I–K). Similarly, in MuSCs overexpressing GFP‐Lin28a, Lin28a, Igf2bp3 and G3bp1 can also simultaneously enrich to form SG (Figure S2A). Altogether, Lin28a and Igf2bp3 respond to stress stimuli, forming SG to regulate the expression of RNAs, including *Igf2bp3* mRNA itself. It can also be seen that the majority of SGs are G3bp1+ Lin28a+ Igf2bp3+ SGs, whilst only a minority lack one of the three components, with G3bp1+ Lin28a+ SGs being the most infrequently observed (Figure 2L), in turn suggesting that Igf2bp3 most likely anchored the formation of Lin28a+ SGs. This is especially interesting, because Igf2bp3 is well‐known to be an m^6^A reader protein.

### 3.3 Lin28a and Igf2bp3 cooperatively enrich the mRNA of DNA damage repair‐related genes

**FIGURE 3:**
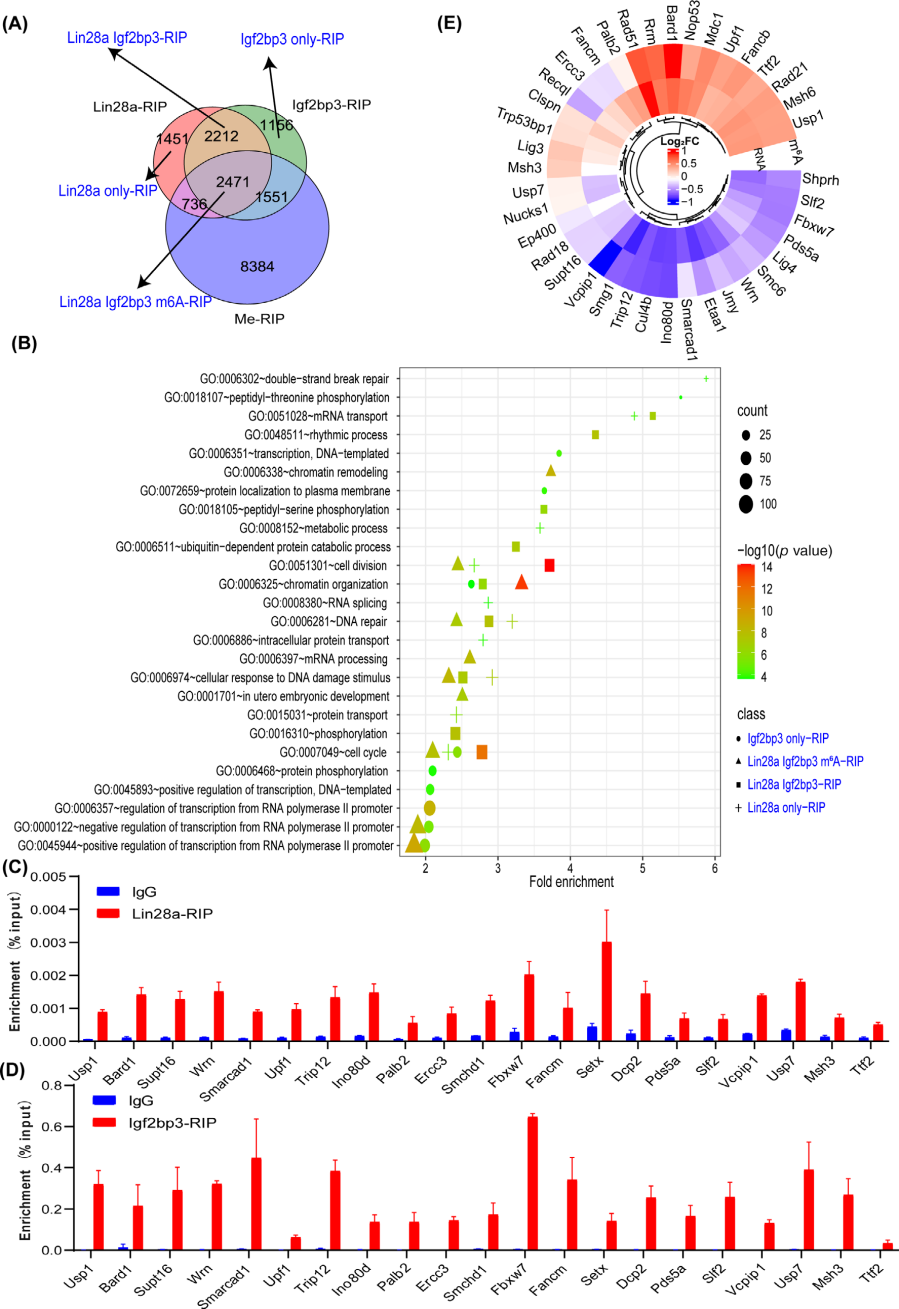
Lin‐28 homologue A (Lin28a) and insulin‐like growth factor 2 mRNA‐binding protein 3 (Igf2bp3) cooperatively enriches the mRNA of DNA damage repair‐related genes. (A) Venn diagram analysis of the Lin28a/Igf2bp3/N6‐methyladenosine (m^6^A)‐RNA immunoprecipitation (RIP)‐seq data. (B) Gene ontology identified the biological processes in which Lin28a/Igf2bp3/m^6^A‐bound transcripts are involved. The top 10 cellular processes of each gene group are plotted. (C) Lin28a‐RIP‐qPCR validated the mRNA enrichment of DNA damage repair‐related genes in Lin28a+ MuSCs. (D) Igf2bp3‐RIP‐qPCR validated the mRNA enrichment of DNA damage repair‐related genes in Lin28a+ MuSCs. (E) Heat map showing the differential expression and m^6^A modification of DNA damage response‐related genes.

To further analyse the regulatory mechanisms of the Lin28a‐Igf2bp3 protein complex on RNA metabolism, we performed RIP‐seq with Lin28a or Igf2bp3 antibodies, MeRIP‐seq with m^6^A antibody and RNA‐seq analysis in control versus Lin28a+ MuSCs. The peaks enriched by RIP‐seq are annotated in Table S3. Venn diagram analysis of the Lin28a/Igf2bp3/m^6^A‐RIP‐seq data showed that 2471 transcripts were co‐bound to Lin28a/Igf2bp3/m^6^A antibodies (Figure 3A). We categorised all enriched transcripts into four groups: (1) transcripts only bound by Lin28a (Lin28a only‐RIP), (2) transcripts only bound by Igf2bp3 (Igf2bp3 only‐RIP), (3) transcripts bound by both Lin28a and Igf2bp3 but not by the m^6^A antibody (Lin28a Igf2bp3‐RIP) and (4) transcripts bound by Lin28a, Igf2bp3 and m^6^A antibodies simultaneously (Lin28a Igf2bp3 m^6^A‐RIP). GO annotation was performed for each of these four groups of transcripts, and the top 10 enriched cellular processes were plotted. The results showed that both the Lin28a Igf2bp3 m^6^A‐RIP group and the Lin28a Igf2bp3‐RIP group were involved in regulating processes such as cell division and DNA damage repair. The Lin28a only‐RIP group of transcripts was primarily involved in cellular processes such as double‐stranded break repair, metabolic processes and cell division. In contrast, the Igf2bp3 only‐RIP group of transcripts was primarily involved in DNA transcription and protein phosphorylation (Figure 3B). We then considered genes significantly enriched in various biological processes from all these groups as the denominator, and calculated the proportion of genes involved in the DNA damage response (DDR). The results revealed that genes enriched in the Lin28a only‐RIP had the highest proportion of DDR genes (26.4%), whereas the Lin28a Igf2bp3 m^6^A‐RIP, Lin28a Igf2bp3‐RIP and Igf2bp3 only‐RIP had 15%–17% DDR genes (Figure S2B).

Given the significant enrichment of DNA repair‐related pathways in transcripts bound by both Lin28a and Igf2bp3, we focused on DNA repair mRNA targets. We validated the enrichment of genes involved in the DNA repair pathway by Lin28a/Igf2bp3‐RIP‐qPCR, and IgG was used as the control. The results showed a significant enrichment of *Usp1*, *Fancm* and *Fbxw7* and other gene transcripts bound by Lin28a and Igf2bp3 (Figure 3C,D). Compared to the control group, we found that these DNA repair gene transcripts have differentially methylated levels at their m^6^A positions and different expression levels (Figure 3E). In summary, our findings suggest that the Lin28a‐Igf2bp3 complex enriches DNA repair‐related transcripts through m^6^A modification, and might promote DNA repair after SG formation in the face of transient cellular stress during MuSCs' self‐renewal.

### 3.4 Lin28a promotes the mRNA stability of DNA damage repair genes and proliferative capacity by enriching m^6^A‐modified transcripts in stress granules

**FIGURE 4:**
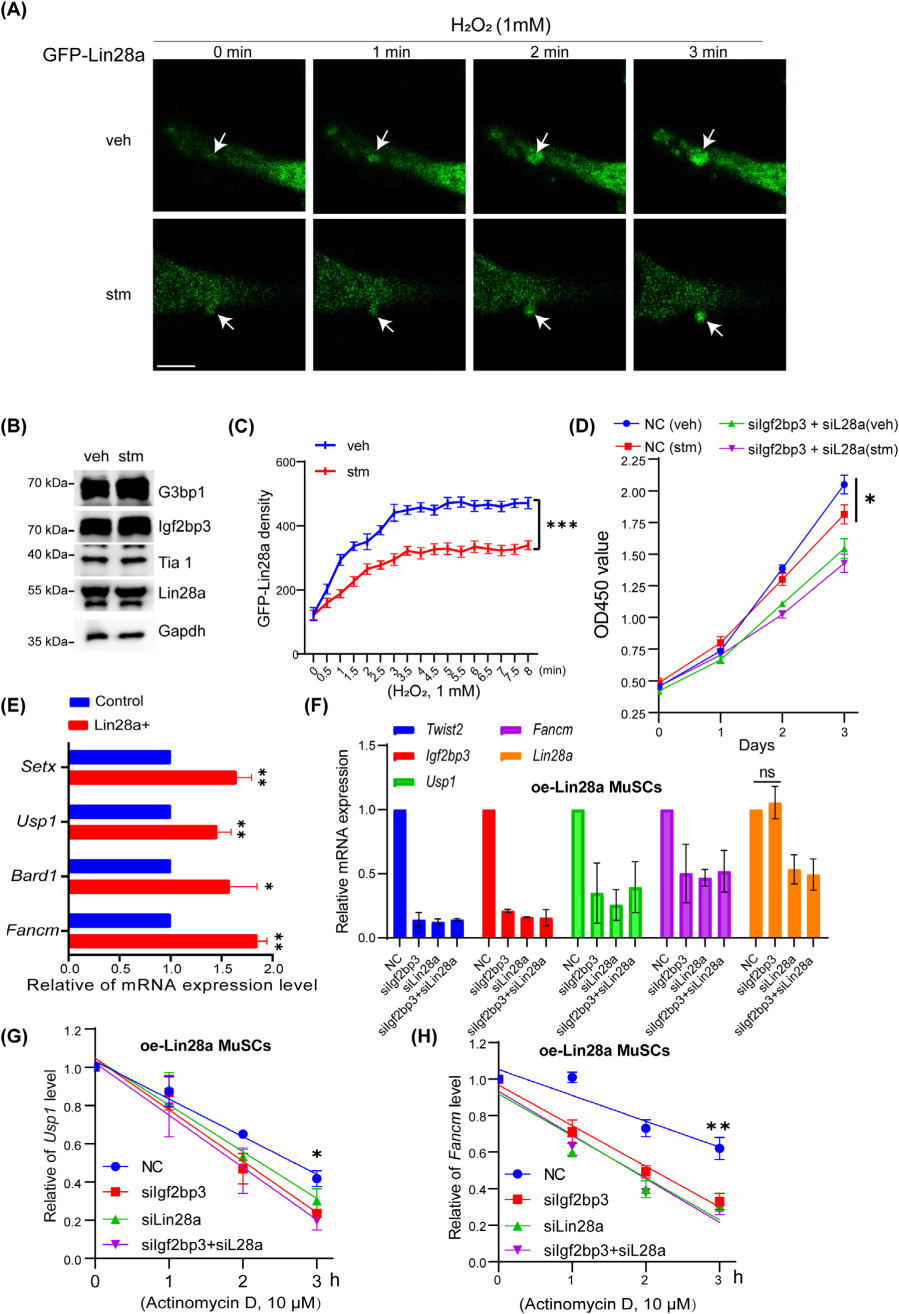
Lin‐28 homologue A (Lin28a) promotes the mRNA stability of DNA damage repair genes and proliferative capacity by enriching N6‐methyladenosine (m^6^A)‐modified transcripts in stress granules. (A) After 24 h of treatment with 20 μM m^6^A inhibitor STM2457 (stm), live‐cell imaging analysis was performed to observe the time and size of GFP‐Lin28a‐stress granules (SG) formation in muscle stem cells (MuSCs) under H_2_O_2_ treatment (1 mM), relative to vehicle treatment (veh). Scale bar: 5 μm. (B) Western blot analysis showed no change of Lin28a‐SG‐associated proteins, such as G3bp1, insulin‐like growth factor 2 mRNA‐binding protein 3 (Igf2bp3) and Tia1, after STM2457 treatment (stm), relative to vehicle treatment (veh). (C) Based on the imaging in (A), the fluorescence density of GFP‐Lin28a‐SG was calculated and compared between the stm and veh groups. The addition of STM2457 significantly reduces the size of GFP‐Lin28a‐SG and also prolongs the formation time. (D) MuSCs treated with STM2457 at 20 μM (stm), followed by CCK8 analysis to assess changes in cell proliferation after 1, 2 and 3 days, relative to vehicle treatment (veh). (E) RT‐qPCR was performed to validate the differential expression of mRNAs, such as *Fancm* and *Usp1*, between the Lin28a+ and control MuSCs groups. (F) RT‐qPCR analysis on MuSCs overexpressing Lin28a, for the expression changes of *Twist2*, *Igf2bp3*, *Usp1*, *Fancm* and *Lin28a* in the group of negative control siRNA (NC), siIgf2bp3, siLin28a or siIgf2bp3 + siLin28a. (G) The mRNA half‐life analysis revealed that under 10 μM actinomycin D treatment, MuSCs overexpressing Lin28a exhibited significant protection against *Usp1* mRNA degradation, whereas knockdown of Igf2bp3 and Lin28a disrupts this protective effect. (H) The mRNA half‐life analysis revealed that under actinomycin D (10 μM) treatment, MuSCs overexpressing Lin28a exhibited significant protection against *Fancm* mRNA degradation, whereas knockdown of Igf2bp3 and Lin28a disrupts this protective effect. Data are presented as means ± SEM. Student's *t*‐test was performed for statistical analysis (*p < 0.05, **p < 0.01 and ***p < 0.001). ns, no significant.

Although the analysis of all the m^6^A‐modified transcripts showed that there were no significant differences in m^6^A modification motifs (Figure S3A), peak distribution (Figure S3B) and the proportion of modified regions in individual RNAs (Figure S3C) in Lin28a+ MuSCs. Compared to the control group, we found that some DNA repair gene transcripts have differentially methylated levels at their m^6^A positions (Figure 3E). According to previous reports, m^6^A modification can promote the formation of SG.^23^ Therefore, we investigated the impact of m^6^A modification level on the specific SG formation of Lin28a with an m^6^A inhibitor (STM2457) treatment, and the duration and size of GFP‐Lin28a‐SG were detected by live‐cell imaging system (Figure 4A). Western blot analysis showed no change in the abundance of Lin28a‐SG‐associated proteins, such as G3bp1, Igf2bp3 and Tia1, before and after STM2457 treatment (Figure 4B). However, analysis of the size and time of GFP‐Lin28a‐SG formation showed that inhibition of mRNA m^6^A modification significantly reduced the SG size and slowed the SG aggregation speed (Figure 4C). Furthermore, STM2457 significantly reduced the proliferation activity of MuSCs, and this inhibitory effect of STM2457 was diminished and not significant when Igf2bp3 and Lin28a were knocked down simultaneously (Figure 4D), indicating that the m^6^A‐dependent proliferative capacity required the Lin28a‐Igf2bp3 complex.

RT‐qPCR was performed to validate the differential expression of mRNAs between the Lin28a+ and control MuSCs groups. The results showed that Lin28a significantly promoted the mRNA expression of DNA damage repair‐related genes such as *Usp1*, *Fancm*, *Setx* and *Bard1* (Figure 4E). In MuSCs overexpressing Lin28a (oe‐Lin28a MuSCs), the expression changes of *Twist2*, *Igf2bp3*, *Usp1*, *Fancm* and *Lin28a* were examined in the negative control siRNA (NC), siIgf2bp3, siLin28a and siIgf2bp3 + siLin28a groups. The results indicated that siLin28a can significantly reduce the mRNA levels of *Igf2bp3*, *Twist2*, *Usp1* and *Fancm*, whilst siIgf2bp3 only decreased the mRNA levels of *Twist2*, *Usp1* and *Fancm* without affecting Lin28a expression, demonstrating the regulatory role of Lin28a in controlling the expression of DNA damage repair proteins Usp1 and Fancm through Igf2bp3 (Figure 4F). The mRNA half‐life analysis revealed that Lin28a can increase the half‐life of *Igf2bp3*, *Usp1* and *Fancm* mRNA (Figures 4G,H and S3D). When Lin28a and Igf2bp3 expression were inhibited by siRNAs, the half‐life of *Usp1* and *Fancm* mRNA significantly decreased in Lin28a‐expressing MuSCs (Figures 4G,H and S3E,F). In conclusion, these results indicate that Lin28a stabilises the mRNA of DNA repair‐related genes by recruiting m^6^A‐modified mRNAs to SG, thus promoting the proliferative capacity of MuSCs.

### 3.5 Lin28a and the m^6^A reader Igf2bp3 promote stress‐induced DNA repair and viability in MuSCs

**FIGURE 5:**
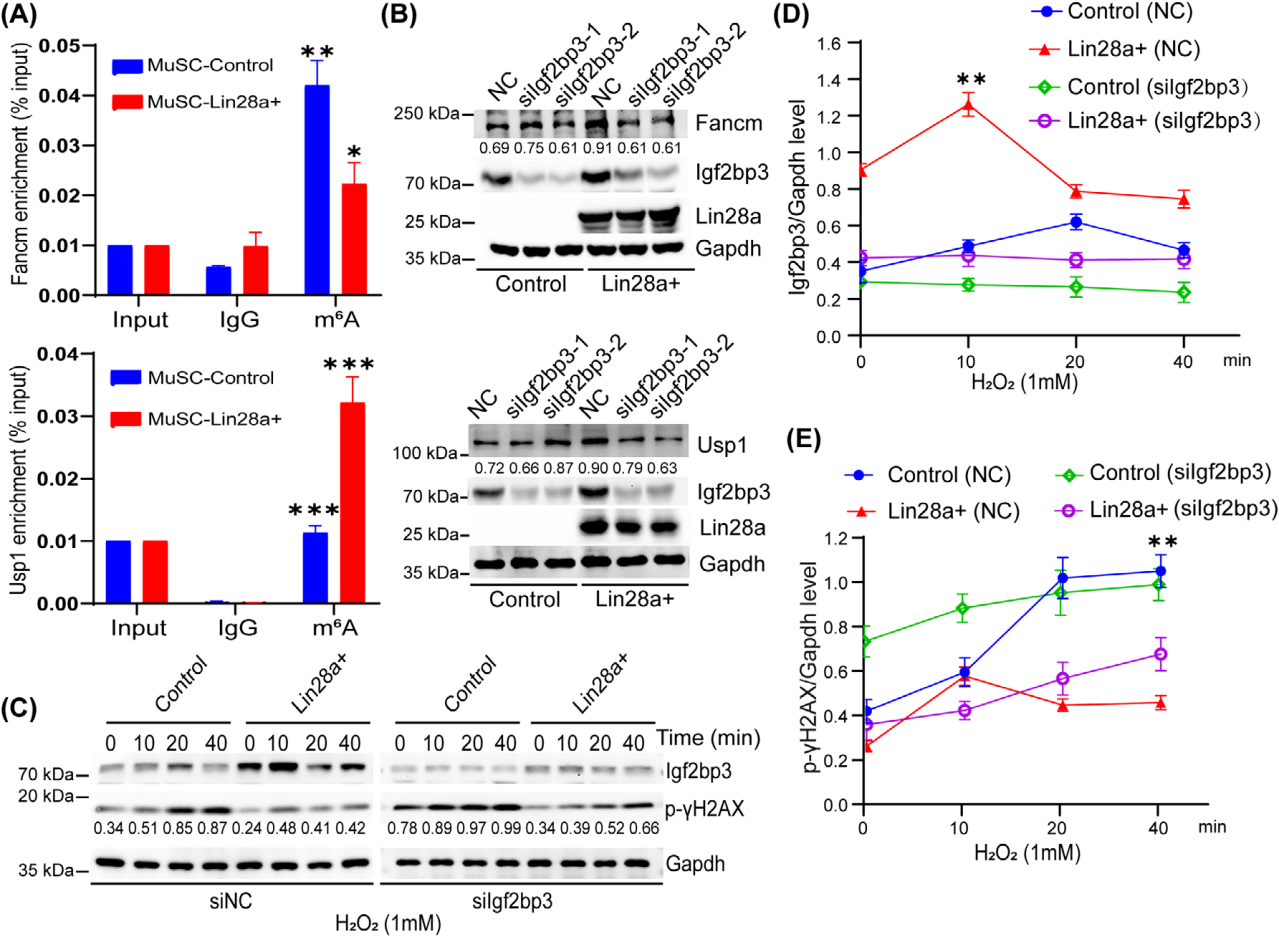
Lin‐28 homologue A (Lin28a) and the N6‐methyladenosine (m^6^A) reader insulin‐like growth factor 2 mRNA‐binding protein 3 (Igf2bp3) promote stress‐induced DNA repair and viability in muscle stem cells (MuSCs). (A) Methylated RNA immunoprecipitation‐qPCR analysis for the abundance of m^6^A modification in *Fancm* and *Usp1* mRNA. (B) Knockdown of Igf2bp3 in Lin28a+ and control MuSCs groups, and Western blot analysis of the protein level of Fancm and Usp1, Gapdh was chosen as the reference protein in this study. (C) Phosphorylation levels of γH2AX (p‐H2AX) were assessed by Western blot, after treating MuSCs with H_2_O_2_ (1 mM) for 10–40 min. (D) Statistical analysis of the grey values of Igf2bp3/Gapdh in (C). (E) Statistical analysis of the grey values of p‐γH2AX/Gapdh in (C). Data are presented as means ± SEM. Student's *t*‐test was performed for statistical analysis (*p < 0.05, **p < 0.01 and ***p < 0.001). NC, negative control siRNA.

In order to confirm the specific mechanism by which Lin28a recruits m^6^A‐modified mRNAs and influences DNA damage repair, we visualised the m^6^A peak enrichment of *Fancm* and *Usp1* in RIP‐seq and MeRIP‐seq data using IGV analysis (Figure S4A), which was also highly consistent with the m^6^A modification sites of Fancm and Usp1 predicted using the SRAMP software (Figure S4B).^35^ The results showed that *Fancm* exhibited the highest m^6^A on exon 14, whilst *Usp1* exhibited the highest m^6^A in the untranslated regions. Such differences in m^6^A modification sites might explain various post‐transcriptional processing events. We designed RT‐qPCR primers around these sites and validated the m^6^A modification levels of *Fancm* and *Usp1* using MeRIP‐qPCR. Compared to IgG, the m^6^A antibody can enrich *Usp1* and *Fancm* mRNA in both control and Lin28a+ MuSCs (Figure 5A). However, the mRNA of *Usp1* is more enriched in Lin28a+ MuSCs, whilst *Fancm* is more enriched in control MuSCs, which is consistent with the sequencing data in Figure 3E. Although *Usp1* and *Fancm* exhibit different m^6^A modification levels in control and Lin28a+ MuSCs, due to the absence of Lin28a expression in the control cells, Lin28a still has a promoting effect on the expression of *Usp1* and *Fancm* in the Lin28a+ cells. Western blot analysis confirmed the upregulation of Usp1 and Fancm by Lin28a, and knocking down Igf2bp3 inhibited the promoting function of Lin28a (Figure 5B).

To functionally validate the effects of the Lin28a‐Igf2bp3 complex on DNA repair in response to oxidative and proteotoxic stress stimuli, we treated MuSCs with H_2_O_2_ or NaAsO_2_, and compared the levels of phosphorylated γH2AX (p‐γH2AX) in the presence of Lin28a overexpression and/or Igf2bp3 knockdown. Western blot results showed that H_2_O_2_ or NaAsO_2_ stimulation of MuSCs increased the phosphorylation level of p‐γH2AX and DNA damage (Figure S5A, left and Figure 5C, left). Lin28a effectively enhanced DNA damage repair capacity and weakened the sharp increase in DNA damage. Lin28a's enhancement of DNA repair was blunted by knocking down Igf2bp3, as evidenced by the sharper increase in DNA damage after siIgf2bp3 (Figure S5A, right and Figure 5C, right). Statistical analysis of experimental replicates' protein data demonstrated that Igf2bp3 was significantly higher in Lin28a+ MuSCs throughout the stress response, and in fact transiently increased further at 10 min upon stimulation with hydrogen peroxide (Figure 5D, red), likely due to the autoregulatory loop between Lin28a and Igf2bp3 (Figure 2B,C), which translated to a reversal of the DNA damage by 20 min (Figure 5E, blue vs. red). Partial knockdown of Igf2bp3 (Figure 5D, violet) led to an abrogation of the DNA damage repair response, as evidenced by the sharper rise in p‐γH2AX (Figure 5E, violet vs. red). Cellular viability analysis indicated that knockdown of Igf2bp3 or Lin28a results in a significant decrease in MuSCs' resistance to the oxidative and proteotoxic stress mediated by H_2_O_2_ or NaAsO_2_ (Figure S5B,C), and knocking down Igf2bp3 also reduced the cell proliferation rate of MuSCs (Figure S5D). Taken together, our results show that the Lin28a‐Igf2bp3 complex in SGs regulates a number of m^6^A‐modified DNA damage repair‐related genes' expression, thereby boosting MuSCs' capacity for stress resistance during self‐renewal and proliferation.

## 4 DISCUSSION

**FIGURE 6:**
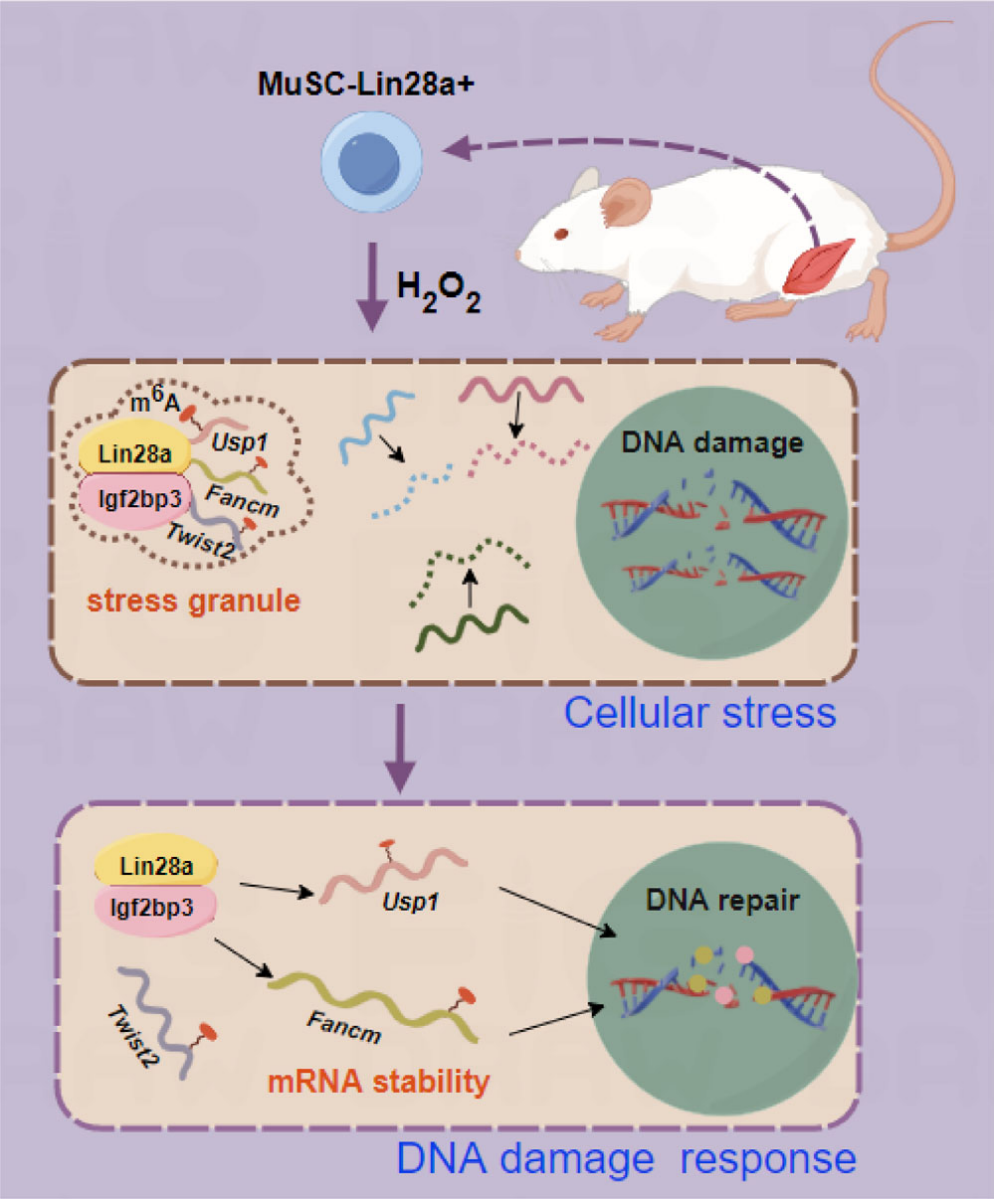
A model diagram representing the interaction of Lin‐28 homologue A (Lin28a) with insulin‐like growth factor 2 mRNA‐binding protein 3 (Igf2bp3) to regulate N6‐methyladenosine (m^6^A)‐modified stress response genes in RNA granules to promote self‐renewal of primary muscle stem cells. MuSCs, muscle stem cells.

In this study, we isolated Lin28a+ MuSCs from mouse muscle tissue and identified the protein factor Igf2bp3 that interacts with Lin28a. Our findings shed light on Lin28a+ Igf2bp3+ granules' RNA‐regulatory mechanisms. In response to acute oxidative and proteotoxic stress, Lin28a+ Igf2bp3+ granules aggregate mRNA of DNA damage repair‐related genes (*Usp1*, *Fancm*, etc.) through their m^6^A modifications, thereby promoting mRNA stability and providing the basis for DNA damage repair during MuSCs' self‐renewal (Figure 6). This molecular mechanism elucidates the important role of Lin28a+ MuSCs in responding to accumulated stress and preventing stem cell senescence. Previous studies have shown that haematopoietic stem cells undergo senescence during ageing due to cumulative DNA damage from oxidative stress.^36^ Moreover, previous studies have shown that DNA repair capacity is crucial for preventing senescence and ensuring the long‐term self‐renewal of multiple tissue stem cell types.^37^ It appears that Lin28a+ MuSCs are shielded from stress damage and senescence, for long‐term self‐renewal, in part through the Lin28a‐Igf2bp3 RNA granules.

Lin28a has been shown in previous studies to play important roles in adult tissue regeneration and energy metabolism.^15^, ^38^ Further investigations have revealed its crucial role in maintaining the self‐renewal of adult tissue stem cells, including a population of MuSCs that exhibited a more primitive and embryonic‐like state.^25^, ^39^ In addition to regulation of target genes through the *let‐7* miRNA system,^40^, ^41^ Lin28a can be packaged into ribonucleoprotein complexes, thus regulating the dormancy versus translation of multiple target gene mRNAs.^42^ Through *let‐7*, Lin28a has been shown to regulate the Igf2bp1–3 family, but not always in tandem, in different cell types.^43^ In Lin28a+ MuSCs, Lin28a significantly increased the mRNA levels of Igf2bp3 and bound to Igf2bp3 protein, but did not affect the expression of Igf2bp1 and Igf2bp2, demonstrating the specificity of regulation between Lin28a and Igf2bp3 in MuSCs. As both Lin28a and Igf2bp3 are mRNA‐binding proteins, we investigated the collaborative regulation of mRNA stability by the Lin28a‐Igf2bp3 complex in MuSCs, and found that they bound and promoted the stability of m^6^A‐modified DNA repair gene mRNAs, and thus DNA repair protein expression during MuSCs proliferation. Whilst previous studies had shown that Lin28a localises to SG in pluripotent stem cells,^44^ their exact function had remained unclear. Our findings indicate that in MuSCs, Lin28a‐Igf2bp3 granules can promote DNA repair capacity in the face of oxidative and proteotoxic stress to ensure the proliferative capacity and self‐renewal of stem cells.

Igf2bp3, a well‐characterised m^6^A reader protein, has been implicated in post‐transcriptional regulation of gene expression.^45^ Previous studies have shown that the phase separation of m^6^A‐modified RNAs is facilitated by proteins with IDR domains like Igf2bp3^16^, ^23^ and, as we have shown, Lin28a. This spatiotemporal control of m^6^A‐modified mRNA stability and expression through Lin28a‐Igf2bp3 granules provides an additional layer of complexity in gene expression regulation. m^6^A modification is a dynamic and reversible RNA modification that plays a crucial role in regulating gene expression.^46^ Interestingly, m^6^A, Lin28 and Igf2bp expression are all required for early embryonic development, and decrease in the later stages of embryonic development,^12^ suggesting that the spatiotemporal control of m^6^A‐modified mRNAs by Lin28a‐Igf2bp3 granules is important to safeguard stem/progenitor cell self‐renewal. In this study, the insignificant expression of Lin28a in the control group does not affect the expression of Usp1 and Fancm. Lin28a obviously increases the expression of Igf2bp3 in Lin28a+ MuSCs. Under stressful conditions, the formation of SG is promoted by m^6^A, increasing the stability of mRNA for *Usp1* and *Fancm*, thereby enhancing DNA damage repair. Whilst Usp1 and Fancm exhibit different m^6^A modifications in control and Lin28a+ MuSCs, Lin28a's role in positively regulating RNA stability via Igf2bp3 is not affected. This explains why Lin28a+ MuSCs have superior stress responsiveness and self‐renewal capacity,^25^, ^47^ that is, to mitigate the oxidative stress and oxidative damage to DNA, especially in stem/progenitor cells and early embryonic development.

Oxidative stress is an imbalance between oxidation and antioxidant activity in the cell, and it is considered to be both a cause and an effect of proteotoxic stress, both of which are significant factors in cellular damage and senescence.^48^ Stress granules are cytoplasmic RNA–protein complexes that form in response to cellular stress (such as oxidative stress, proteotoxic stress, etc.), regulating the translation and degradation of mRNAs in tandem with cell cycle control.^49^ Recent studies suggest that certain membrane‐less organelle proteins or granules of cellular macromolecules undergo separation‐aggregation via phase separation mechanisms at different stages of the cell cycle to facilitate cell cycle progression.^50^ Moreover, reversible aggregation protects Cdc19 from stress‐induced degradation, and several enzymes required for the G1 phase can reversibly aggregate during transient stress to allow for a quick restart of the cell cycle after stress,^51^ and DHX9 SGs shield daughter cells from RNA damage originating from parental cells.^52^ Similarly, our study found that Lin28a forms granules with Igf2bp3 in the cytoplasm, to store m^6^A‐modified mRNAs involved in mitochondrial TCA cycle, oxidative stress response, DNA damage repair and so on (Figure 3B). This mechanism also explains why Lin28a+ MuSCs are more resistant to stress, pass through the G1 cell cycle phase more quickly (Figure 1E,F), and ensure the transfer of DDR‐related RNAs to daughter cells in an embryonic‐like manner.

In conclusion, we have found that Lin28a upregulates the expression of Igf2bp3 and interacts with Igf2bp3 protein. The Lin28a‐Igf2bp3 complex regulates m^6^A‐modified mRNAs, forming RNA granules that can undergo phase separation and respond to cellular stress stimuli with enhanced DNA repair capacity. This is beneficial for the proliferation of MuSCs and might play an important role in maintaining the long‐term self‐renewal capacity of muscle stem cells.

## AUTHOR CONTRIBUTIONS

DS designed and performed the experiments, and drafted the manuscript. YC, PW and YC helped with the experiments. NS‐C designed and supervised the entire project.

## CONFLICT OF INTEREST STATEMENT

The authors declare no conflicts of interest. Ng Shyh‐Chang is an Editorial Board member of Cell Proliferation and a co‐author of this article. He was excluded from the editorial decision‐making related to the acceptance of this article for publication in the journal.

## Supporting information


**FIGURE S1.** Lin28a promotes MuSCs proliferation. (A) Analysing the correlation between m^6^A modification abundance and transcript expression levels in MuSCs (Lin28+ vs. control), Pearson's *R* = 0.89. Generating heatmaps of transcripts related to DNA repair (B), cell cycle (C) and oxidative phosphorylation (D). CCK8 analysis demonstrates that (E) overexpression of Lin28a promotes MuSCs proliferation. (F) Knockdown of Lin28a reduces the proliferation rate of MuSCs. Data are presented as means ± SEM. Student's *t*‐test was performed for statistical analysis (*p < 0.05).

**FIGURE S2.** The localization and functional analysis of stress granules of Lin28a, Igf2bp3 and G3bp1. (A) Co‐localization analysis of Lin28a, Igf2bp3 and G3bp1 in stress granules. Scale bar: 10 μm. (B) Analysis of the proportion of DDR‐related genes in GO‐annotated biological processes.

**FIGURE S3.** Analysing MeRIP data, the mRNA half‐life of Usp1 and Fancm. (A) Homer‐annotated motif of the m^6^A‐modified sequences in MuSCs. (B) Distribution of m^6^A peaks in different regions of mRNA. (C) Proportion of m^6^A peaks in different regions of mRNA. (D) The mRNA stability of *Igf2bp3* after overexpression of Lin28a in MuSCs. (E, F) The mRNA stability of *Usp1* and *Fancm* after knockdown of Igf2bp3 in Lin28a+ MuSCs.

**FIGURE S4.** Analysis of the abundance of Fancm and Usp1 peaks and prediction of m^6^A modification. (A) IGV visualisation of the enrichment peaks of m^6^A/Lin28a/Igf2bp3‐RIP on *Fancm* and *Usp1* mRNAs. (B) SRAMP prediction of m^6^A modification sites on *Fancm* and *Usp1* mRNAs.

**FIGURE S5.** Lin28a and Igf2bp3 promote cell viability. (A) After treating MuSCs with NaAsO_2_ (500 μM) for 40 min, the changes in phosphorylation levels of γH2AX (p‐H2AX) were assessed by Western blot. (B, C) In MuSCs, knockdown of Igf2bp3 and Lin28a, combined with treatment with H_2_O_2_ or NaAsO_2_, resulted in a significant decrease in cell viability by CCK8 analysis. (D) Knocking down Igf2bp3 reduced the cell proliferation rate of MuSCs. Data are presented as means ± SEM. Student's *t*‐test was performed for statistical analysis (*p < 0.05, **p < 0.01).

**Table S1.** The primers used for quantitative analysis.

**Table S2.** The list of genes related to Lin28A expression.

**Table S3.** RIP‐peaks annotation.

## Data Availability

The data that support the findings of this study are openly available in GSE262720 at https://www.ncbi.nlm.nih.gov/geo.
