# Supplementary figures and images for: Lin28a forms an RNA‐binding complex with Igf2bp3 to regulate m6A‐modified stress response genes in stress granules of muscle stem cells
Source: Cell Prolif. 2024 Jul 17;57(12):e13707. doi: 10.1111/cpr.13707 (PMC11628740; doi:10.1111/cpr.13707)

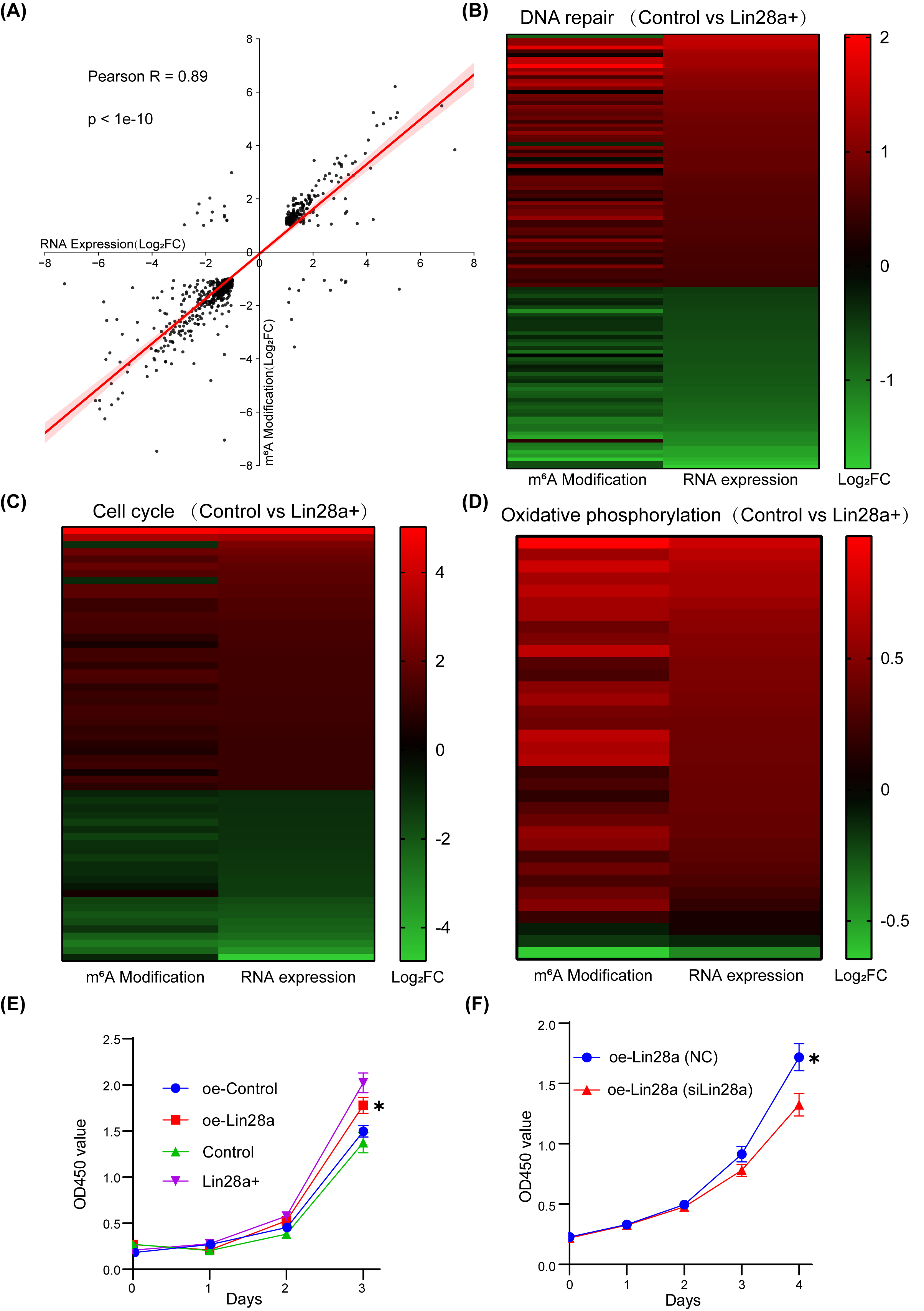

Supplement: Supplementary file 1 — FIGURE S1. Lin28a promotes MuSCs proliferation. (A) Analysing the correlation between m6A modification abundance and transcript expression levels in MuSCs (Lin28+ vs. control), Pearson's R = 0.89. Generating heatmaps of transcripts related to DNA repair (B), cell cycle (C) and oxidative phosphorylation (D). CCK8 analysis demonstrates that (E) overexpression of Lin28a promotes MuSCs proliferation. (F) Knockdown of Lin28a reduces the proliferation rate of MuSCs. Data are presented as means ± SEM. Student's t‐test was performed for statistical analysis (*p < 0.05). [file CPR-57-e13707-s001.tif]

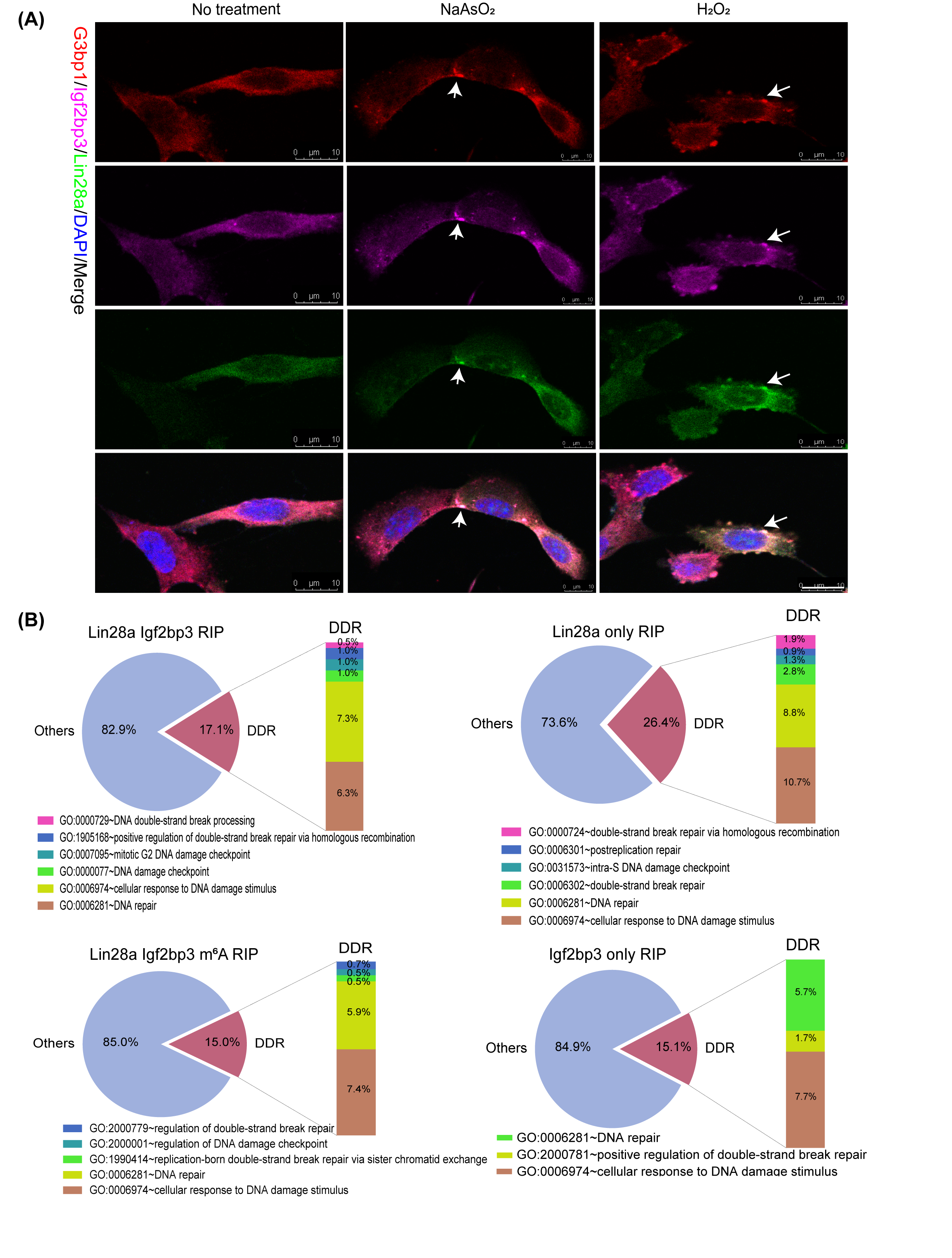

Supplement: Supplementary file 2 — FIGURE S2. The localization and functional analysis of stress granules of Lin28a, Igf2bp3 and G3bp1. (A) Co‐localization analysis of Lin28a, Igf2bp3 and G3bp1 in stress granules. Scale bar: 10 μm. (B) Analysis of the proportion of DDR‐related genes in GO‐annotated biological processes. [file CPR-57-e13707-s003.tif]

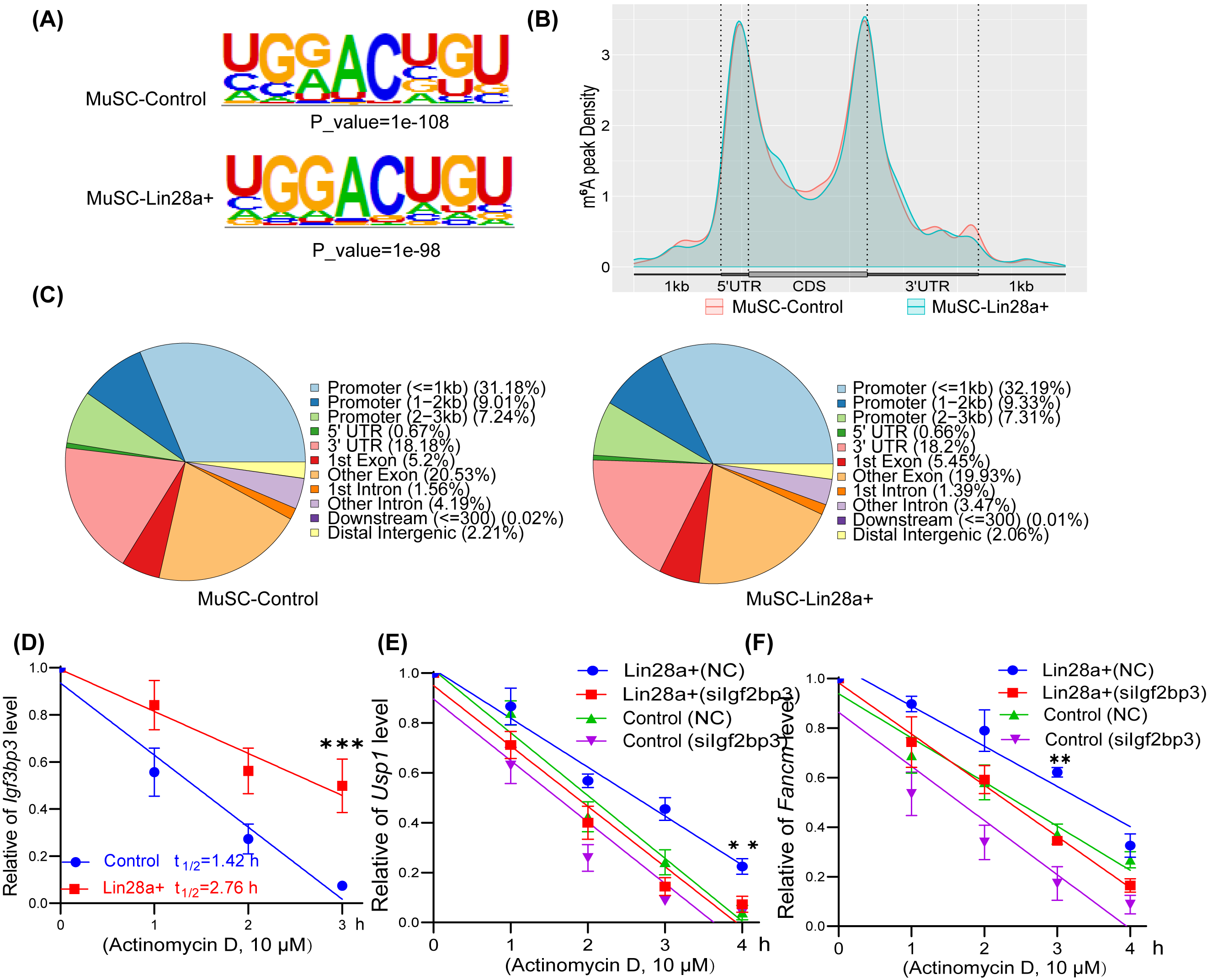

Supplement: Supplementary file 3 — FIGURE S3. Analysing MeRIP data, the mRNA half‐life of Usp1 and Fancm. (A) Homer‐annotated motif of the m6A‐modified sequences in MuSCs. (B) Distribution of m6A peaks in different regions of mRNA. (C) Proportion of m6A peaks in different regions of mRNA. (D) The mRNA stability of Igf2bp3 after overexpression of Lin28a in MuSCs. (E, F) The mRNA stability of Usp1 and Fancm after knockdown of Igf2bp3 in Lin28a+ MuSCs. [file CPR-57-e13707-s002.tif]

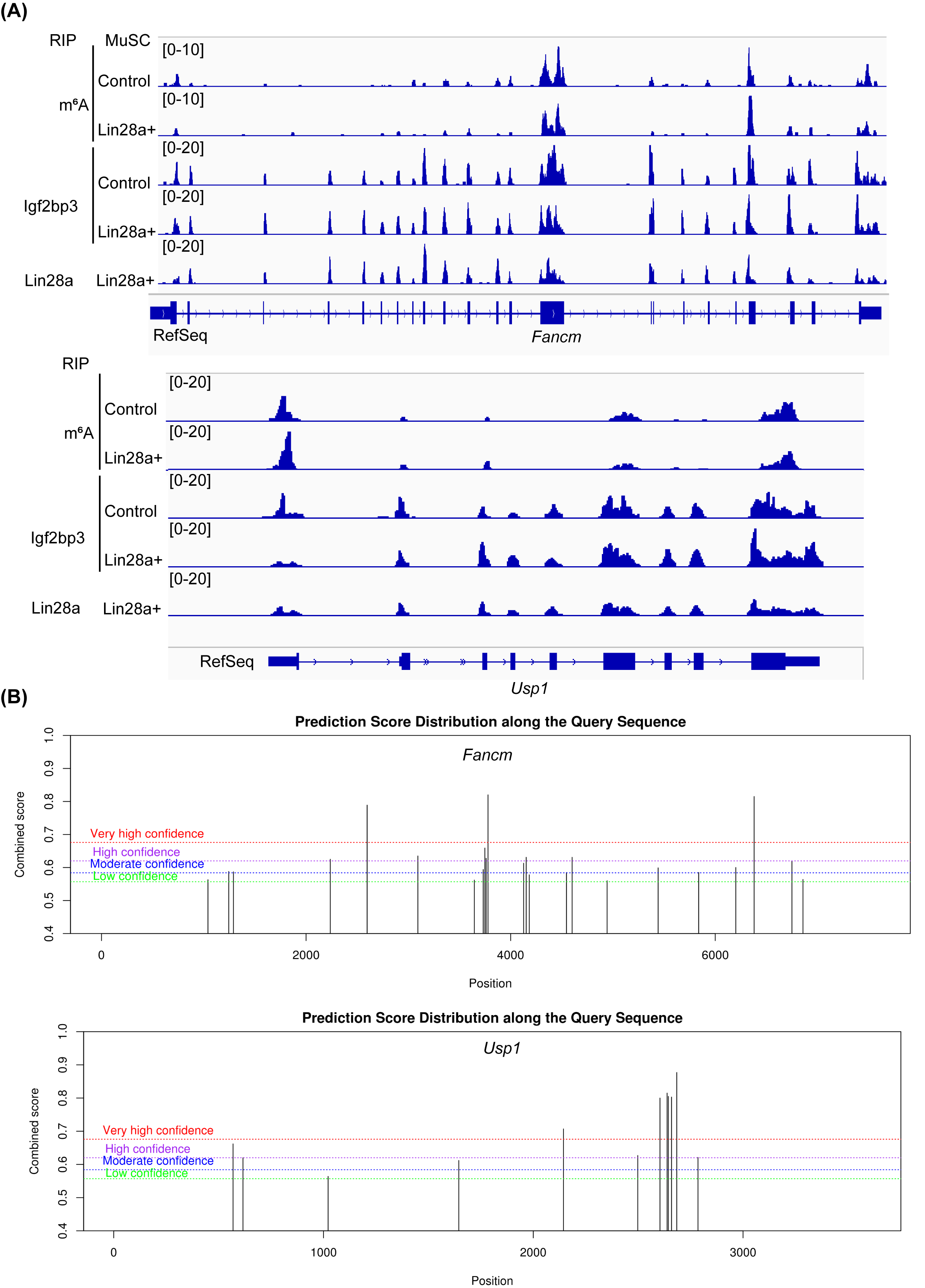

Supplement: Supplementary file 4 — FIGURE S4. Analysis of the abundance of Fancm and Usp1 peaks and prediction of m6A modification. (A) IGV visualisation of the enrichment peaks of m6A/Lin28a/Igf2bp3‐RIP on Fancm and Usp1 mRNAs. (B) SRAMP prediction of m6A modification sites on Fancm and Usp1 mRNAs. [file CPR-57-e13707-s006.tif]

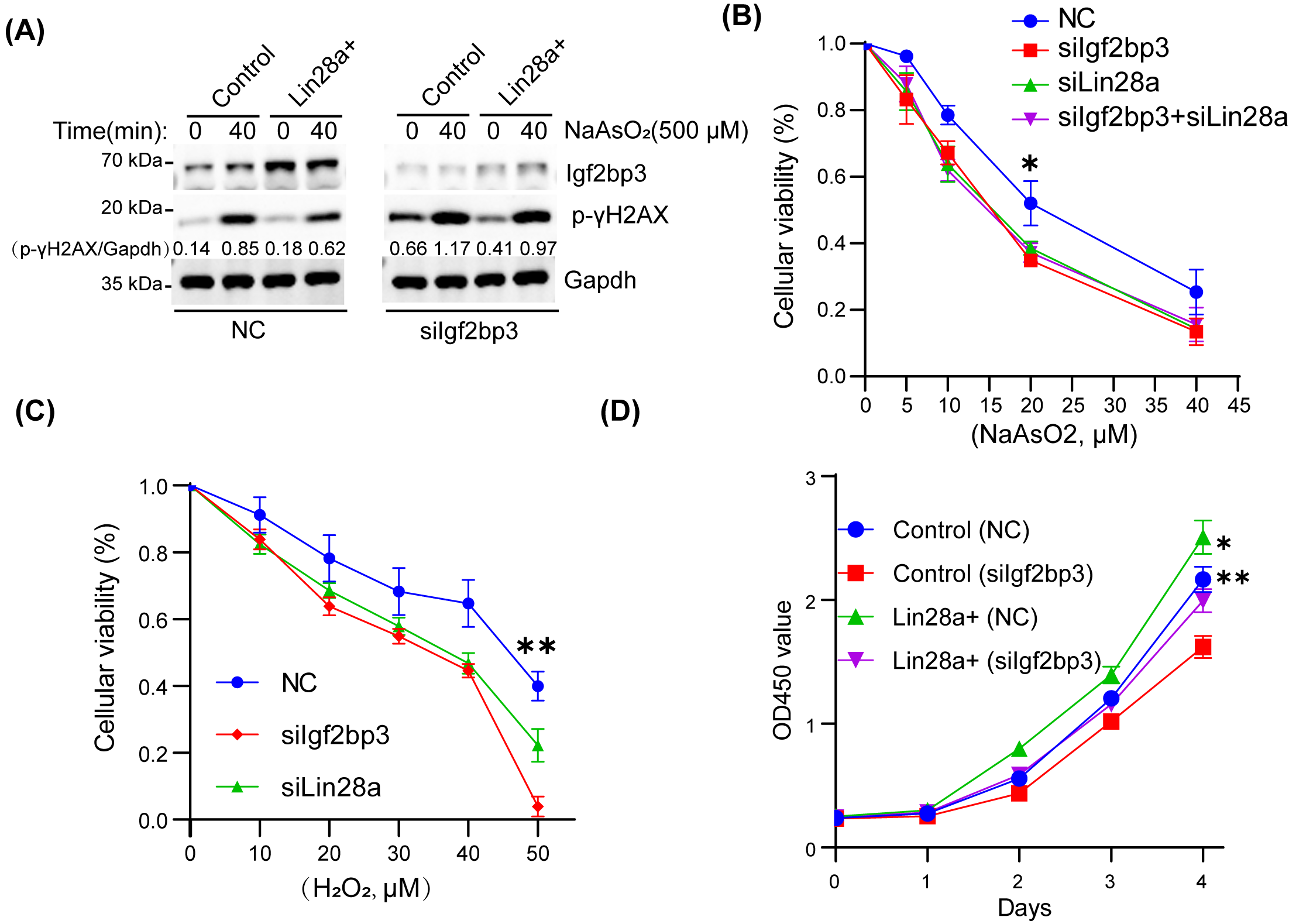

Supplement: Supplementary file 5 — FIGURE S5. Lin28a and Igf2bp3 promote cell viability. (A) After treating MuSCs with NaAsO2 (500 μM) for 40 min, the changes in phosphorylation levels of γH2AX (p‐H2AX) were assessed by Western blot. (B, C) In MuSCs, knockdown of Igf2bp3 and Lin28a, combined with treatment with H2O2 or NaAsO2, resulted in a significant decrease in cell viability by CCK8 analysis. (D) Knocking down Igf2bp3 reduced the cell proliferation rate of MuSCs. Data are presented as means ± SEM. Student's t‐test was performed for statistical analysis (*p < 0.05, **p < 0.01). [file CPR-57-e13707-s004.tif]
